# Free Energy Profile of APOBEC3G Protein Calculated by a Molecular Dynamics Simulation

**DOI:** 10.3390/biology1020245

**Published:** 2012-07-26

**Authors:** Yoshifumi Fukunishi, Saki Hongo, Masami Lintuluoto, Hiroshi Matsuo

**Affiliations:** 1 Biomedicinal Information Research Center (BIRC), National Institute of Advanced Industrial Science and Technology (AIST), 2-3-26, Aomi, Koto-ku, Tokyo 135-0064, Japan; Email: y-fukunishi@aist.go.jp; 2 Division of Applied Life Science, Graduate School of Life and Environmental Science, Kyoto Prefectural University, Kyoto 606-8522, Japan; Email: s_hongo@mei.kpu.ac.jp (S.H.); masami@kpu.ac.jp (M.L.); 3 Department of Biochemistry, Molecular Biology and Biophysics, University of Minnesota, Minneapolis, MN 55455, USA

**Keywords:** APOBEC3G, GBSA, molecular dynamics simulation, free energy surface, Protein structure, NMR, HIV-1

## Abstract

The human APOBEC3G protein (A3G) is a single-stranded DNA deaminase that inhibits the replication of retrotransposons and retroviruses, including HIV-1. Atomic details of A3G’s catalytic mechanism have started to emerge, as the structure of its catalytic domain (A3Gctd) has been revealed by NMR and X-ray crystallography. The NMR and crystal structures are similar overall; however, differences are apparent for β2 strand (β2) and loops close to the catalytic site. To add some insight into these differences and to better characterize A3Gctd dynamics, we calculated its free energy profile by using the Generalized-Born surface area (GBSA) method accompanied with a molecular dynamics simulation. The GBSA method yielded an enthalpy term for A3Gctd’s free energy, and we developed a new method that takes into account the distribution of the protein’s dihedral angles to calculate its entropy term. The structure solved by NMR was found to have a lower energy than that of the crystal structure, suggesting that this conformation is dominant in solution. In addition, β2-loop-β2’ configuration was stable throughout a 20-ns molecular dynamics (MD) simulation. This finding suggests that in solution A3Gctd is not likely to adopt the continuous β2 strand configuration present in the APOBEC2 crystal structure. In the NMR structure, the solvent water accessibility of the catalytic Zn^2+^ was limited throughout the 20-ns MD simulation. This result explains previous observations in which A3G did not bind or catalyze single cytosine nucleotide, even when at excessive concentrations.

## 1. Introduction

Human APOBEC3G protein (A3G) is a prominent member of a multifunctional family of Zn^2+^-dependent polynucleotide cytidine deaminases (e.g., reviewed by [1,2,3]). This family was named after APOBEC1 (apolipoprotein B mRNA editing enzyme catalytic polypeptide 1), which edits *APOB* mRNA C6666 to generate an early stop codon and a shorter polypeptide. AID also belongs to this family, which edits immunoglobulin gene DNA cytidines to trigger somatic hypermutation and class switch recombination. A3G and six other human APOBEC3 proteins have been shown to inhibit the replication of a variety of retrotransposons and retroviruses. Importantly, A3G can restrict HIV-1, the causative agent of AIDS, by using its catalytic function [4,5,6,7]. A3G incorporation into virions in producer cells and its delivery to target cells are required. During reverse transcription in target cells, A3G deaminates cytidine to uridine within newly transcribed minus-strand viral DNA (e.g., reviewed by [1,2,3]). Subsequently, adenine replaces guanine in plus-strand DNA resulting in G-to-A hypermutation, which can in turn inactivate the viral genome (e.g., reviewed by [1,2,3]). All members of this family contain a highly conserved Zn^2+^-binding motif with the amino-acid consensus sequence His-X-Glu-X_23-28_-Pro-Cys-X_2-4_-Cys (where X stands for any amino acid). APOBEC3B, APOBEC3D/E, APOBEC3F and A3G contain two Zn^2+^-binding motifs, however only the C-terminal one is catalytically active. 

Based on crystal structures of bacterial and yeast cytidine deaminases [8,9,10], a possible mechanism of deamination was proposed. A histidine and two cysteine residues bind catalytic Zn^2+^, while a glutamic acid residue interact with Zn^2+^ through a water molecule. Zn^2+^ activates the bound water molecule creating a hydroxide group that makes a nucleophilic attack on C4 of the primidine ring. This nucleophilic attack changes the atomic orbital configuration of C4 from sp^2^ to sp^3^, prompting release of the amino group.

Structures of the A3G catalytic domain (A3Gctd) have been reported; three were solved by NMR structures (2JYW [11], 2KBO [12] and 2KEM [13]) and two by X-ray crystallography (3IR2 [14] and 3E1U/3IQS [15]). 2JYW, 2KEM and 3IR2 contain mutations to increase solubility while retaining its deamination activity and HIV-1 restriction. 2KBO and 3E1U/3IQS (3E1U has been updated and re‑deposited as 3IQS) use protein samples of wild-type amino acid sequence. There are slight differences in the protein lengths, with 2KEM being the longest spanning amino acids 191–384 and 3E1U/3IQS the shortest, spanning 197–380. The overall secondary and tertiary structure of these solved structures are similar, each containing five beta-strands and six alpha-helices. 

Although, structural features of A3Gctd have emerged, there is no structure of the ssDNA-A3G complex. ssDNA-binding surfaces of A3Gctd have been suggested based on NMR titration experiments and computer modeling, in which the binding path lies along a line connecting Arg313 and the catalytic Zn^2+^ [11,12,13]. Another DNA-binding pathway has been proposed based on a crystal structure (3E1U/3IQS), in which ssDNA binds a pathway that spans Arg374 and the catalytic Zn^2+^, and then kinks toward Asn244 [15]. A common feature of both pathways is that the ssDNA binds surfaces created by loops including loop207–219, loop244–257 and loop313–320. Loop207–219 and loop244–257 are close to the catalytic site in 2JYW and 2KEM, whereas they are remote from the catalytic site in 3E1U/3IQS. These differences in the loop configurations led to the two different models for DNA-binding [11,12,15]. 

**Figure 1:**
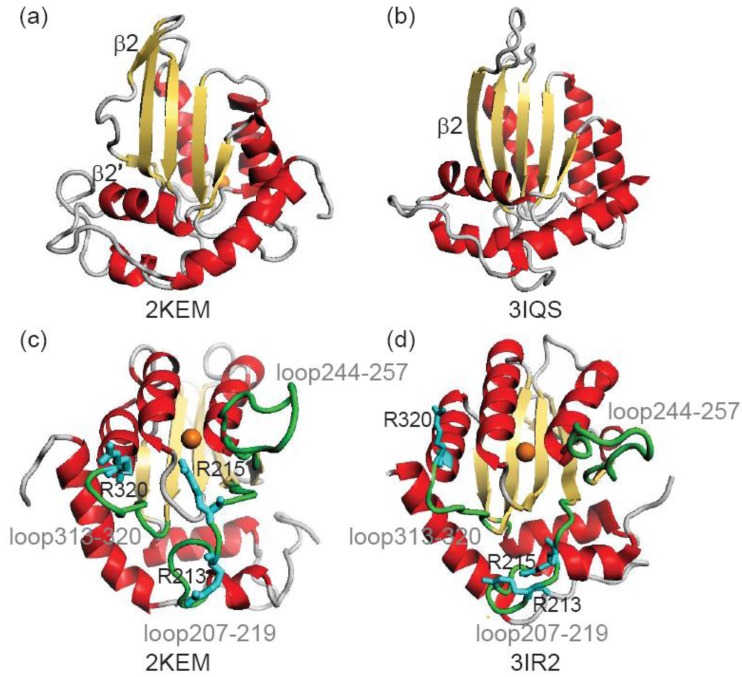
Comparison of an NMR structure (2KEM) and crystal structures (3IR2 and 3IQS)of A3Gctd. (**a**) and (**b**) show the β2-loop-β2’ structure and a continuous β2 structure, respectively. (**c**) and (**d**) shows the diffence in the positions of loops, loop207–219, loop244–257 and loop313-320 (green), and arginines, Arg213, Arg215 and Arg320 (cyan). Orange sphere represents catalytic Zn^2+^ ion.

β2-loop-β2’ structure is a unique feature of A3Gctd, as in other members of the cytidine deaminase family this strand is continuous (Figure 1a). The β2-loop-β2’ structure of A3Gctd implies that A3Gctd does not form an inter-domain or inter-molecular β-sheet using this region, yet many researchers have been using full-length A3G structural models in which the N-terminal domain (A3Gntd) is connected with A3Gctd through extensive inter-domain β2-β2 interactions. Such models were generated by using the APOBEC2 crystal structure, which is reported to be a homo-dimer with the two monomers interacting through an inter-molecular β-sheet formed by the two β2 strands [16]. APOBEC2 contains only one Zn^2+^ binding domain, allowing its homo-dimeric structure be used to generate full-length structural models of A3G. In order to generate such APOBEC2-based A3G models, the β2-loop-β2’ structure of A3Gctd was unfolded and then rearranged to be extended. In a recent MD study by Autore *et al*., the authors suggested that the β2-loop-β2’ structure became the continuous β2 strand in water solvent at room temperature (Figure 1b) [17]. This directly conflicts with NMR data, which were collected in water solvent at room temperature [11,12,13]. 

To examine the stability of A3Gctd structure we performed 20-ns MD simulations of an NMR structure (2KEM) and a crystal structure (3IR2). The flexibility of loops and arginines that are important for DNA-binding was calculated by using trajectories of MD simulations. In addition, the free energies of 2KEM and 3IR2 were calculated using the Generalized-Born Surface Area (GBSA) method [18,19,20,21,22,23,24,25] to determine which structure is more populated in solution at room temperature. An improved version of the AMBER force field (FUJI force field) was used for the MD simulation. Further more, possible structural-transition pathways between 2KEM and 3IR2 are revealed by using targeted MD simulations.

## 2. Methods

We calculated the free energy difference between an NMR structure (2KEM) and a crystal structure (3IR2). These two structures were chosen because they were determined in similar buffer conditions and with the same expression and purification conditions. Both 2KEM and 3IR2 have the same β2-loop-β2’ structure, while loop207–219, loop244–257 and loop313–320 show differences. Free energy profiles were calculated along pathways converting from 2KEM to 3IR2 as well as from 3IR2 to 2KEM. 

A3Gctd structures were solvated with a water sphere during the MD simulations. We applied an NVT MD simulation with sphere system, which gives canonical ensemble (Helmholtz free energy) by conserving moles (N), volume (V) and temperature (T). We used a repulsive-potential wall of the sphere that allowed the volume change of the system. The free energy calculation was performed in vacuum by the GBSA method. The most important thing in free-energy calculation is the realization of canonical ensemble; the probability is given by the Boltzmann distribution. The integrator applied in this study (Hoover–Evans method) can realize the canonical ensemble. It has been suggested that the GBSA approximation may not provide quantitatively accurate results in the estimation of distribution probability although it gives qualitatively accurate results [25]. Thus, our analysis can tell which structure is more stable in solution, while the analysis cannot calculate quantitatively accurate distribution probability of structures.

The outline of the procedure for the free-energy calculation is as follows.

**Step 1a.** 2KEM and 3IR2 structures were energy-minimized, then subjected to a short-time MD simulation, which generated an equilibrium structure for each of 2KEM and 3IR2.
**Step 2a.** Ten long-time (20 ns) MD simulations were performed with different initial velocities for the equilibrium structures of 2KEM and 3IR2. 20 trajectory structures were extracted from each 20-ns MD simulation.
**Step 3a.** The average and deviation of free energy of the extracted trajectory structures were calculated by using the GBSA method.

The outline of the procedure to calculate the free-energy profile is as follows.

**Step 1b.** Method described in Step 1a.
**Step 2b.** Targeted MD simulations from 2KEM to 3IR2 and from 3IR2 to 2KEM were calculated, which generated intermediate structures along each pathway [26]. Ten structures were sampled with similar intervals for Step 3.
**Step 3b.** MD simulations (10 ns) were performed to generate the structural ensemble of each structure sampled in the Step 2b. The coordinates of main-chain atoms of each sampled structure were kept by using weak constraints in explicit solvent molecules. Two MD simulations were performed with different initial velocities for each sampled structure.
**Step 4b.** Average GBSA energy was calculated based on its atomic coordinate trajectory found in the MD simulations performed in the Step 3b.
**Step 5b.** The free energy profile was calculated based on the GBSA energy for each path of the targeted MD simulations.

The details of MD simulations are explained in the Section 2.1.

### 2.1. Molecular Dynamics Simulation in Water Solvent

Atomic coordinates of A3Gctd structures were downloaded from the Protein Data Bank 2KEM (NMR structure) and 3IR2 (crystal structure). Both structures have mutations (L234K, C243A, F310K, C321A and C356A), which were manually replaced with their wild-type amino acid by using the Spanner homology modeling program [27]. These initial structures were superimposed by the program GASH prior to MD simulations [28].

**Figure 2:**
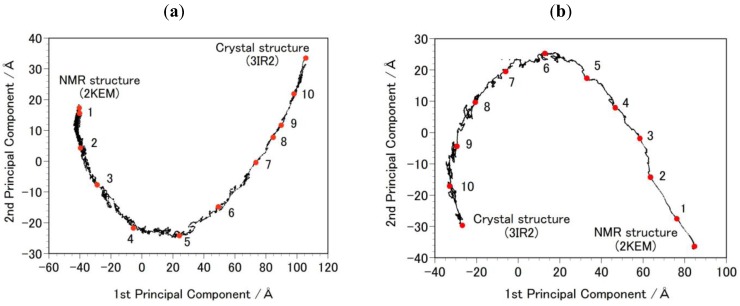
Principal Component Analysis (PCA) result of the targeted molecular dynamics (MD) simulation. Each black dot in the figure represents one intermediate structure of the protein during the targeted MD. The red-filled circles, numbered 1 to 10, indicate structures selected for the free-energy profile calculation (Figure 3). The results were projected onto PCA1 and PCA2 axes. (**a**) shows the path from 2KEM to 3IR2. Trajectory structures were numbered in ascending order. (**b**) shows the path from 3IR2 to 2KEM, and trajectory structures were numbered in descending order.

**Step 1a:** An equilibrium structure was generated for 2KEM and 3IR2, and MD simulations performed in explicit water system. The atomic charges and force field originated from FUJI, which is a modified version of AMBER parm99 [29,30]. The systems were solvated in a water sphere with a radius of 40 Å. The protein structure was embedded in TIP3P [31] water with ion particles included Cl^−^ to neutralize the total charge of the system. After a steepest descent (1000 steps) energy minimizations with positional restraints on the solute and to carry out an initial 100 ps simulation with the positions of the solute atoms restrained by a force constant of 10 kcal/mol Å^2^ to let the water diffuse around the molecule and for equilibration. The fast multipole method (FMM) [32] was used for the calculation of electrostatic contribution to non bonded interactions with a cut-off of 12 Å. The MD simulations were performed by using the cosgene/myPresto program [33,34].
**Step 2a:** The NVT calculation (20 ns) at 300K was performed. The position restraint was not applied to any atom. The SHAKE algorithm was applied to the system and the time step was set to 2 fs [35]. Snap shot structures were obtained every 10 ps during the NVT run, obtaining 2000 snap shot structures for each initial structure. The solvent molecules were removed from these structures and free energy was calculated by using the GBSA method.
**Step 2b:** Targeted MDs were performed to generate a free energy profile from the NMR structure (2KEM) to the crystal structure (3IR2) as well as a free energy profile from the crystal structure to the NMR structure [26]. Computational conditions were the same as that used for the Step 1a, except for an additional force pulling protein main-chain atoms from the initial structure to the final structure. The additional force was parabolic with a force constant of 0.01–0.15 kcal/mol/Å^2^. The total simulation time was 2 ns and the protein structures were sampled every 5 ps. The sampled structures were analyzed by the Principal Component Analysis (PCA) of the protein atomic coordinates. PCA extracts major structural changes of an ensemble of protein structures. Figure 2 shows PCA of the structures obtained from the targeted MDs. Each dot represents one structure and the averaged structure is set to the origin of the axes. The axis in PCA corresponds to the normal modes of the structural changes. The reaction pathways are continuous and smooth which indicates that there is no significant energy jump during the MD simulation, therefore the target MD has successfully shown a possible path of the structural changes between the NMR and the crystal structures. Ten structures were selected with the same intervals in order to calculate free energies in Step 3b.
**Step 3b: **MD simulations of the sampled structures in Step 2b were performed in the solvent water as described in Step 1a. After the energy minimization and the equilibration run described in step1a, the NVT production run (1 ns) at 300 K was performed. To keep the protein structure, the position restraint was applied to the main-chain atoms with a force constant of 0.01 kcal/(mol Å^2^). The SHAKE algorithm was applied to the system and the time step was set to 2 fs [35]. Snap shot structures were obtained every 10 ps, which resulted in collecting 1000 snap shot structures for each sampled structure. The solvent molecules were removed from these snap shot structures and the GBSA method was applied to calculate free energy in Step 4b.

**Figure 3:**
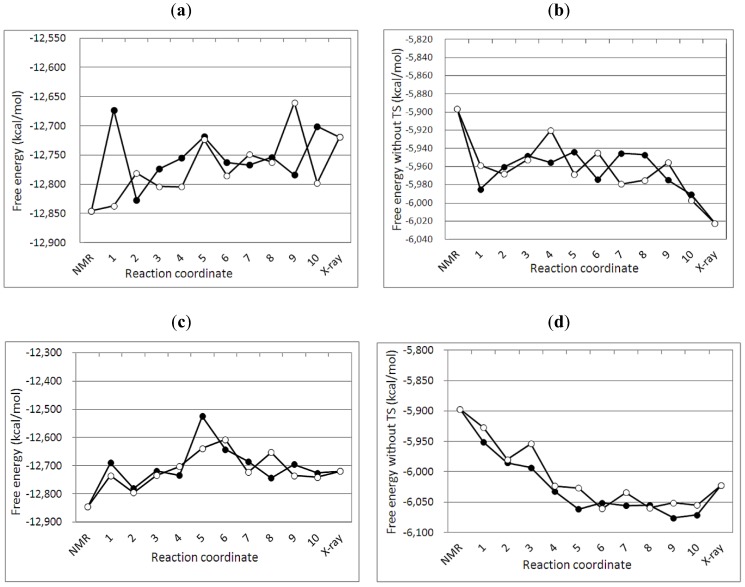
Free energy profiles of targeted MDs between the NMR and the crystal structures of A3Gctd. (**a**) and (**b**) show free energy G1(with entropy) and G2(without entropy) profile, respectively, along the reaction pathfrom the NMR (2KEM) to the crystal (3IR2) structure. Numbers in the X axis correspond to the numbers (selected structures) in Figure 2a. (**c**) and (**d**) show free energy G1(with entropy) and G2(without entory) profile, respectively, along the pathfrom the crystal to the NMR structure. Numbers in the X axis correspond to the numbers (selected structures) in Figure 2b.Two MD simulations were performed with different initial velocities for each path way, and filled circles and open circles represent the data obtained from the first and the second MD simulations, respectively.

### 2.2. GBSA Calculation

The free energy was calculated by the GBSA method to compare the relative stability of A3Gctd structures. Instead of the explicit water model, the GBSA implicit solvent model was applied to describe the solvent [18] by using the Hawkins formulation in the GB part [23] with the conventional surface area calculation with a probe radius of 1.4 Å [36]. The other GBSA parameters were the same as those used in the previous paper [25]. The GBSA calculations were performed by using the program cosgene/myPresto. In conventional GBSA method, the interactions among protein atoms and water solvent molecules (enthalpy term) and the entropy term of water solvent are taken into account. The entropy of protein is not taken into account in the conventional GBSA method, and it must be added to calculate free energy of proteins. 

### 2.3. Newly Developed Method to Calculate Protein Entropy

Usually, the entropy of protein can be calculated based on normal mode analysis. But in the current case, the protein has two stable forms and the intermediate structures are not energy minima. The normal mode analysis is not an adequate method for such a double minima potential, thus a new method of the calculating entropy of a protein was needed. Entropy of a protein includes the entropy terms for bond, angle and torsion (dihedral angle). The bond entropy, including C-H, O-H, N-H and S-H, and angle entropy, including H-X-H (X=C, N, O and S), are small because bond lengths and angles are fixed to constant values in MD simulations. The entropy of torsion angles contributes to the free energy since torsion angles freely rotate in the MD simulation.

Our calculated entropy for protein dihedral angles was described by Equation (1).


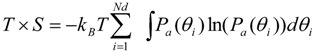
(1)

here P_a_, θ_i_ and Nd are the probability of dihedral angle of the a-th form, the i-th dihedral angle and the total number of dihedral angles, respectively. The total free energy of the a-th form is given by


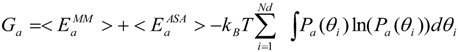
(2)

where <E_a_^MM^> and <E_a_^ASA^> represent the averaged GB and ASA terms of the a-th form, respectively. The GB term consists of the bond, angle, torsion, inter-atomic interaction energies obtained by the classical force field and the GB electrostatic energy. We examined the TS value by changing the number of bins from 12 degrees to 120 degrees. The TS values with number of bins of 12, 30, and 60 degrees were similar to each other, whereas that of 120 degrees was different. Thus, the number of bins for integration of Equation 1 was set to 30 degree. In the current study, Ga value [Equation (2)] and the Ga value without the TS term are denoted as G1 (with entropy) and G2 (without entropy), respectively.

## 3. Results and Discussion

### 3.1. Stability of Structures

We examined the stability of the NMR (2KEM) and crystal (3IR2) structures of A3Gctd by using MD simulations. Ten MD simulations for 20 nanoseconds with different initial velocities for each structure (2KEM or 3IR2) were performed in Step 2a. The RMSD of main chain atoms after 10 ns and 20 ns are summarized in Table 1 and Table 2, respectively. The two data sets are very similar, indicating that the structures reached their equilibrium states after 10 ns of MD. After 20 ns of MD, the average RMSD for main chain atoms from their pre-MD state is 2.64 Å and 2.04 Å for the NMR and crystal structures, respectively. Moreover, the RMSDs after 20 ns MD for the NMR and crystal structures in comparison to the pre-MD crystal and NMR structures respectively is 4.16 Å and 3.97 Å. Therefore differences between the NMR and crystal structures were maintained after 20 ns of MD. In both cases, the β2-loop-β2’ structure was maintained after 20 ns MD. 

**Table 1:** RMSD of main-chain atoms in Å unit after 10-ns MD simulations.

No of MD simulation	MD simulation of the crystal structure			MD simulation of the NMR structure		
	X-ray *vs.* X-ray ^a^	X-ray *vs.* NMR ^b^	No of ligand H_2_O	NMR *vs.* NMR ^b^	NMR *vs.* X-ray ^a^	No of ligand H_2_O
1	2.22	3.36	0.43	2.21	4.02	0.30
2	1.63	3.75	0.32	3.75	4.13	0.33
3	1.93	4.31	0.34	1.86	3.87	0.23
4	2.11	3.91	0.49	2.47	4.41	0.42
5	1.70	4.07	0.46	2.62	4.56	0.46
6	1.72	3.96	0.27	2.76	4.52	0.44
7	2.38	3.41	0.46	2.76	3.90	0.42
8	2.27	3.56	0.29	2.29	4.72	0.28
9	1.88	4.10	0.57	3.27	3.84	0.33
10	1.97	3.80	0.58	2.59	4.00	0.28
Average	1.98	3.82	0.42	2.66	4.20	0.35

^a^ the RMSD values of the trajectories against the crystal structure; ^b^ the RMSD values of the trajectories against the NMR structure.

**Table 2:** RMSD of main-chain atoms in Å unit after 20-ns MD simulation.

No of MD simulation	MD simulation of the crystal structure			MD simulation of the NMR structure		
	X-ray *vs.* X-ray ^a^	X-ray *vs.* NMR ^b^	No of ligand H_2_O	NMR *vs.* NMR ^b^	NMR *vs.* X-ray ^a^	No of ligand H_2_O
1	1.85	3.59	0.43	2.21	4.02	0.30
2	1.96	4.09	0.32	3.75	4.13	0.52
3	2.23	4.29	0.34	1.86	3.87	0.22
4	2.13	4.34	0.50	2.28	4.05	0.49
5	1.80	3.85	0.46	2.62	4.56	0.25
6	2.48	3.78	0.27	2.76	4.52	0.33
7	2.77	3.64	0.46	2.76	3.90	0.32
8	1.82	3.82	0.30	2.29	4.72	0.31
9	1.58	4.13	0.57	3.27	3.84	0.32
10	1.82	4.18	0.58	2.59	4.00	0.31
Average	2.04	3.97	0.43	2.64	4.16	0.34

a the RMSD values of the trajectories against the crystal structure; b the RMSD values of the trajectories against the NMR structure.

Arg 213, Arg215 and Arg320 are important for DNA binding (Figure 1) [11] and the main chain RMSD of these arginines before and after 20 ns MD are summarized in Table 3. Arg213 and Arg215 exhibited RMSDs of 3.95 Å and 4.24 Å respectively in the NMR structure comparison and 3.38 Å and 2.60 Å respectively in the crystal structure comparison. These values are greater than the average RMSD (Table 1 and Table 2), suggesting Arg213 and Arg215 move more than other A3Gctd residues during the MD simulations. The three-dimensional configuration of Arg213 and Arg215 differ between the NMR and crystal structures, and differences between these structures were maintained after 20-ns MD with RMSD values for post-MD NMR *vs.* pre-MD crystal structures of 8.20 Å (Arg213) and 8.64 Å (Arg215) and post-MD crystal *vs.* pre-MD NMR of 8.42 Å (Arg213) and 8.35 Å (Arg215). Arg 213 and Arg215 are located within loop207–219, which exhibits an average RMSD of 1.18Å and 1.23Å for the crystal and NMR structure, respectively. RMSDs for loop244-257 and loop313-320 were also calculated, with values of 2.46Å (crystal) and 2.48Å (NMR) for loop244–257, and 1.13Å (crystal) and 1.35 Å (NMR) for loop313–320. These results suggest that loops 207–219 and 313-320 are less flexible in solution compared to loop244–257, which is consistent with NMR structures of A3Gctd in which loop207–219 and loop313–320 are better defined than loop244–257 [11,13].

**Table 3:** Main-chain RMSD of three arginines after 20-ns MD simulation.

Arginines	NMR *vs.* NMR ^a^	NMR *vs.* X-ray ^b^	X-ray *vs.* X-ray ^c^	X-ray *vs.* NMR ^d^
Arg213	3.95	8.20	3.38	8.42
Arg215	4.24	8.64	2.60	8.35
Arg320	2.29	2.24	1.53	1.95

RMSD is in Å; ^a^ the RMSD values of the trajectories of MD simulation of the NMR structure against the initial NMR structure; ^b^ the RMSD values of the trajectories of MD simulation of the NMR structure against the initial crystal structure; ^c^ the RMSD values of the trajectories of MD simulation of the crystal structure against the initial crystal structure; ^d^ the RMSD values of the trajectories of MD simulation of the crystal structure against the initial NMR structure.

### 3.2. Free Energy Differences between the NMR and Crystal Structure

Free energy values were calculated for ten trajectory structures sampled in the 20 ns MD simulations; these values are summarized in Table 4. Ten calculations were performed for each trajectory structure with different initial velocities, and the deviation (σ) of the resulting ten energy values was calculated. If the G1 value was calculated by Equation (2) including the entropy of the protein, the NMR structure was about 100 kcal/mol more stable than the crystal structure, and this energy difference was larger than one standard deviation. Without the protein entropy term (G2), the crystal structure was about 100 kcal/mol more stable than the NMR structure. This result indicates a greater range of dihedral angles in the NMR structure compared to the crystal structure, suggesting that the amino acids are more loosely packed in solution compared to in the crystal. The free energy values suggest that the NMR structure (2KEM) is more stable than the crystal structure (3IR2) in water. It is reasonable that an enthalpy-driven structure was determined by using the crystallized form of A3Gctd because the entropy of a protein approaches zero when crystallized. 

**Table 4:** Free energy and protein entropy term obtained from 20-ns MD simulation.

No of MD simulation	Crystal structure 3IR2			NMR structure 2KEM		
	G2	TS	G1	G2	TS	G1
1	−6043.13	−6702.60	−12745.74	−5884.37	−7027.53	−12911.90
2	−6036.39	−6736.90	−12773.30	−5944.13	−7121.26	−13065.39
3	−6044.5	−6847.83	−12892.33	−5923.86	−6868.51	−12792.37
4	−6047.56	−6750.79	−12798.36	−5939.21	−7080.32	−13019.53
5	−6024.11	−6719.73	−12743.84	−5936.12	−6916.54	−12852.66
6	−6065.13	−6664.80	−12729.94	−5895.72	−6944.52	−12840.24
7	−6034.97	−6781.37	−12816.35	−5935.67	−6947.93	−12883.60
8	−6040.02	−6734.99	−12775.01	−5915.02	−6966.65	−12881.67
9	−6026.65	−6828.27	−12854.92	−5941.27	−6922.34	−12863.61
10	−6036.85	−6849.60	−12886.46	−5900.79	−6926.11	−12826.90
Average	−6039.93	−6761.69	−12801.62	−5921.62	−6972.17	−12893.79
σ	10.94	60.04	56.22	20.37	75.38	81.28

The unit is kcal/mol.

### 3.3. Reaction Mechanism

A3Gctd contains one zinc atom (Zn^2+^) in its catalytic center, which can bind a water molecule that attacks the target cytosine for deamination. The number of contacting water molecules to the catalytic Zn^2+^ was examined in the trajectory structures of the MD simulations (Table 1 and Table 2). On average, the zinc atom of the NMR structure contacts 0.34 ± 0.07 water molecules whereas in the crystal structure, it contacts 0.43 ± 0.10 water molecules. This result suggests that the catalytic Zn^2+^ is more accessible to water in the crystal structure than in the NMR structure. In the NMR structure, the position of loop207–219 contributes to limiting solvent accessibility of the catalytic Zn^2+^ (Figure 1), and MD simulations showed that loop207–219 maintained its positions in solution. If the catalytic Zn^2+^ is less accessible to solvent water, it is also less accessible to the target cytosine, which is consistent with the observations that neither cytosine nor cytidine could bind A3G, despite A3Gctd sharing all essential residues of the catalytic site with other single-nucleoside or single-nucleotide cytosine deaminases [11,37]. 

### 3.4. Free Energy Profile between the NMR and Crystal Structures

The free energy profile of the targeted MD simulation from the NMR (2KEM) to the crystal (3IR2) structure was qualitatively similar to that of the crystal to NMR structure (Figure 3). In both pathways, the NMR structure had a lower free energy than the crystal structure (G1 values in Figure 3a,c). The crystal structure exhibits lower free energy than the NMR structure when the entropy of the protein is not considered (G2 values in Figure 3b,d). Interestingly, there is an energy barrier in the free energy profiles, which indicates that both 2KEM and 3IR2 structures are at a local minimum. 

## 4. Conclusions

We calculated the free energy of A3Gctd by combining the GBSA method with MD simulations and developed a new method for calculating free-energy in which the entropy of the protein is estimated based on the distribution of its torsion angles. The MD simulation showed that the NMR structure (2KEM) has a lower free energy than the crystal structure (3IR2), suggesting that the NMR structure is dominant in water. There is an energy barrier between 2KEM and 3IR2, indicating that the transition from the entropically stable NMR structure to the enthapically stable crystal structure is difficult at room temperature over a short period of time; this finding is consistent with the results of the 20 ns MD simulations. 

The β2-loop-β2’ structure was stable in the 20 ns MD simulation as it was maintained in all trajectory structures. The structure of the β2 region is important because researchers of A3G have been building structural models based on the APOBEC2 crystal dimer in which two monomers of APOBEC2 are connected by a β-sheet that consists of β2 strands from each monomer [16]. Autore *et al*., suggested that the results of their MD simulation, in which the β2-loop-β2’ becomes one extended β2 strand would be consistent with the assembly of the N-terminal domain of A3G (A3Gntd) and A3Gctd through β2-β2 interactions, thus resembling the APOBEC2 dimeric structure. A recently published paper by Krzysiak *et al.* showed that APOBEC2 is a monomer in solution, and suggesting that the intermolecular β-sheet formed through β2-β2 interactions is a crystallographic artifact [38]. Our results offer further evidence that the β2-loop-β2’ is maintained and does not become a single β-strand to form an inter-domain β-sheet with A3Gntd.

Loop207–219 maintains its position during the 20 ns MD simulation thus keeping access to the catalytic Zn^2+^restricted in the NMR structure (2KEM). The interaction of Arg 215 and Arg213 with a ssDNA substrate may change the position of loop207–219 allowing the target cytosine to bind to the catalytic site. We look forward to an A3Gctd-ssDNA complex structure to resolve this debate on how A3Gctd binds to its substrate.
